# Microstructural response and wear behaviour of Ti-6Al-4V impregnated with Ni/Al_2_O_3_ + TiO_2_ nanostructured coating using an electric arc

**DOI:** 10.1038/s41598-022-25918-4

**Published:** 2022-12-20

**Authors:** Kavian Cooke, Abdulrahman Alhubaida

**Affiliations:** Faculty of Engineering and Informatics, University of Bradford, Richmond Road, Bradford, West Yorkshire UK

**Keywords:** Engineering, Materials science

## Abstract

Titanium alloys are known for their excellent corrosion resistance; however, low surface hardness results in poor wear resistance, which limits its potential application. This study employs a novel two-step process to embed a hard Ni coating containing a mixture of nanosized particles (Al_2_O_3_ and TiO_2_) into the surface of the Ti-6Al-4V alloy using an electric arc produced during the inert tungsten gas welding process. The surface of the sample was evaluated using Vickers Microhardness, Scanning electron microscopy, Energy dispersive spectroscopy and pin-on-plate wear testing. Microstructural analysis showed that impregnating the titanium surface with Ni/(Al_2_O_3_ and TiO_2_) nanomaterials resulted in the formation of a hard martensitic structure to a depth of approximately 2 mm below the surface. The changes observed are driven by modification of the surface chemistry and the presence of nickel, causing grain size reduction, solid solution strengthening and dispersion strengthening of the treated layer by the nanoparticles. The hardness of the treated layer increased by more than 180% when 40 nm Al_2_O_3_ and 30 nm TiO_2_ particles were embedded into the surface. Similarly, the wear resistance of the treated surface improved by 100%.

## Introduction

The use of titanium alloys has substantially expanded since their first development in the early 1950s. It now finds application in several extreme work environments where high strength and performance are required^1^. The mechanical and corrosion resistance properties are desirable and have significant applications in the automotive, aerospace and biomedical industries^2^. Considerable attention has also been devoted to titanium alloys in different fields, including military gadgets and civilian products. The first practical titanium alloy was Ti6Al4V alloy, developed in the 1950s for aerospace and military applications. Many years after its development, the Ti6Al4V alloy is still the most successful and frequently used material in biomedical, aerospace applications^3^.

Although Ti6Al4V alloy possesses many desirable mechanical strength characteristics and corrosion resistance, a primary limitation is low surface hardness resulting in poor wear resistance and high friction coefficient^4^. These limitations prevent the application of Ti6Al4V alloy in situations where high contact loads are used^4^. Over the last two decades, numerous methods of improving the surface hardness of titanium alloys have been investigated, such as ion implantation^5^, thermal treatments, physical vapour deposition (PVD), and chemical vapour deposition (CVD)^6^. Gas nitriding has demonstrated the most significant promise to enhance the hardness of the surface layer through a high-temperature diffusion process typically carried out in the region of 1000 °C^7^^,^^8^. A limiting factor in this process is the grain growth consistently recorded due to high-temperature exposure^9^. The combined gas and CVD process has also shown the potential for enhancing the hardness of the titanium surface. However, the process is discontinuous since it has to be carried out in two reactors^10^. In another study by Tobola et al.^11^, the researchers explored a two-step process in which titanium components were burnished with a force of 130 N before being subjected to a gas nitriding process. While the surface hardness of the Ti6Al4V increased, the mechanical treatment led to the formation of many defects in the form of dislocation and grain boundary openings. Techniques such as surface coatings have been attempted; however, a primary limitation of this method is poor adhesive strength between the deposited coatings and the titanium alloy^12^.

Other techniques involve using concentrated energy sources to harden the surface of the titanium alloy. Typical energy sources include laser^13^, plasma or electron beam to provide the high-power density required to treat the surface^14^. Though these techniques show significant potential for hardening titanium alloy surfaces, the equipment needed is prohibitively expensive. In another study, the surface of the Ti6Al4V surface was hardened using the electric arc generated during inert tungsten gas welding to melt the surfaces in conjunction with nitrogen gas to produce a nitride layer on the surface of the Ti6Al4V alloy. The area that has been heat-treated is generally covered with nitrogen to produce a nitride layer. Argon gas is also used to prevent any form of contamination. The hardness and wear resistance of the nitrided layers depended on the density and quantity of the nitrogen gas used. Improving the surface properties of a material by modifying the surface has become an essential requirement before any practical tribological application^15^. Ti6Al4V alloy requires an appropriate surface treatment to improve its performance in friction reduction, hardness, resistance to wear, and chemical stability^8^^,^^10^. Surface treatment ensures that Ti6Al4V alloy retains its bulk desired properties while expanding its application in various fields. Surface modification is also a good factor that encompasses the performance of an engineering component and its cost. Different surface modification technologies of Ti6Al4V alloy have been developed based on their chemical properties. These techniques have shown various levels of success and additional limitations stemming from the cost of equipment used and the time consumed in achieving surface changes and improvements for wear resistance^4^^,^^5^.

TIG welding is used in thermal oxidation. All these processes involve the diffusion of interstitial atoms. TIG welding is preferred in the surface treatment of Ti6Al4V alloy because it possesses some desirable characteristics. For instance, it can provide concentrated heating of the piecework. It has an inert gas that protects the weld pool by shielding it. The filler material is not an essential requirement in TIG welding, and when needed, the filler material does not require fine preparation. Also, it does not result in slag formation; hence, treatment of the weld is unnecessary.

This article proposes a novel two-step process that involves the deposition of a thin ultrahard particle-reinforced nanocrystalline coating followed by melting the coated surface of the Ti-6Al-4V alloy using an electric arc generated by a tungsten electrode in the TIG welding process. The method simplifies the embedding/integration of hard nanoparticles into surface Ti-6Al-4V to enhance wear resistance. It utilises a readily available heat source that can be found in most job shops while lending itself to flexible applications in several industries. The melting and impregnation of the treated layer diversifies the microstructural features which increases the surface hardness that can address various applications with different requirements for wear resistance and mechanical properties.

## Materials and methods

The Ti-6Al-4V samples were cut to a dimension of 10 × 20 × 5 mm using a band saw. Three specimens were tested for each condition. The samples were prepared by grinding progressively on abrasive paper to 1200 grit size and then polished using a particle-impregnated paste of 6 µm and 1 µm diameter, respectively. The samples were cleaned in an ultrasonic acetone bath for fifteen minutes following the polishing step.

### Modification of the surface of the titanium alloy

**Figure 1:**
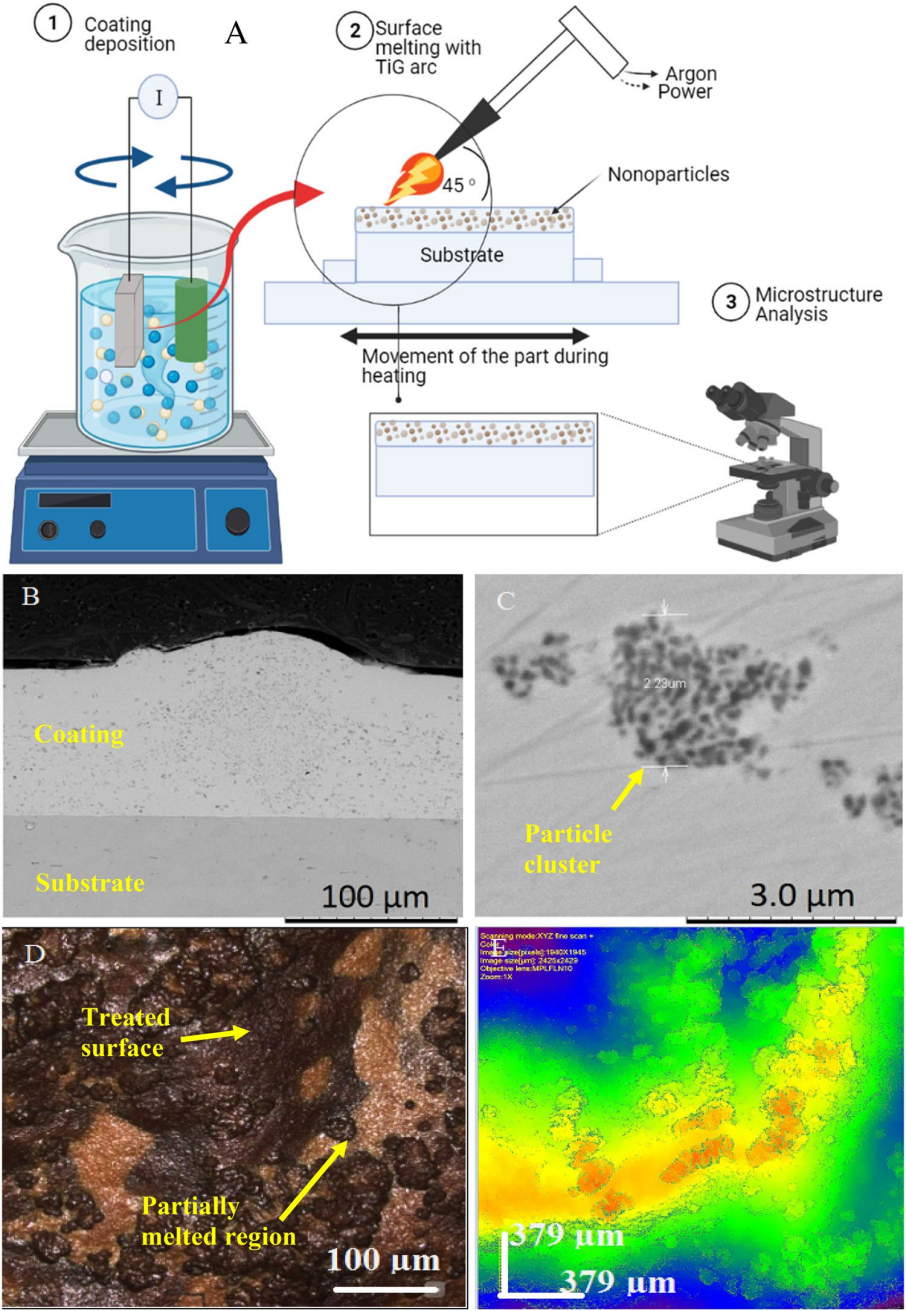
Experimental setup. (**A**) Schematic surface treatment process (**B**) shows an example of the coating deposited onto the titanium. (**C**) Particle cluster within the deposited layer (**D**) Texture of the melted surface (**E**) Confocal map of the surface shows the height variation in the surface.

Each polished sample was coated with Ni coating deposited from a modified Watts Nickle bath solution^16^^,^^17^. The electrodeposition process was carried out in a 250 ml glass beaker. A schematic of the coating process is shown in Fig. 1A. Two baths were prepared with the following constituents 250 g/L NiSO_4_.6H_2_O, 45 g/LNiCl_2_.6H_2_O, 35 g/L H_3_BO_3_, 1 g/L Saccharin, 1L H_2_O^18^. The following mixture of nanoparticles was added to each solution: Bath-1 (20 g/L) 40 nm-Al_2_O_3 _and (20 g/L) 250 nm TiO_2_ nanoparticles and Bath-2 (20 g/L) of 40 nm-Al_2_O_3 _and 250 nm TiO_2_ nanoparticle. The coating parameters such as current density of 5 A/dm^2^ and a deposition temperature of 50 °C were optimised in a previous study^16^. The surface of the coated samples was melted using a tungsten electrode with a voltage of 100 V, a traverse speed of 2 mm/s and Argon (99.98% purity) as the shielding gas (see Fig. 1A. The electrode was positioned at 45° to the workpiece and 1 mm above the titanium surface to be melted.

Each sample was prepared by three passes to ensure complete melting of the sample surface. The specimen's surfaces have been welded using a 2% thoriated electrode at 75 Amp. The samples were fixed to a steel platform to ensure all samples were the same distance away from the torch, which was 2 mm. Table 1 summarises the testing parameters that were used in the study. Microstructural analysis and hardness testing samples were cut transverse to the melted surface and mounted in Bakelite. The surfaces were prepared as outlined above and etched with Kroll's reagent. The micro-hardness of the treated region was evaluated using a Vickers micro-hardness tester with a 0.2 kg load, according to ASTM E384. A dry sliding wear test was also performed at room temperature using a reciprocating pin-on-plate machine, according to ASTM G133 standard. The tribological tests were performed on a custom reciprocating pin-on-plate tribometer. The titanium surfaces were tested against a diamond pin used as the counter-face material under a constant load of 50 N and a sliding velocity of 0.75 m/s for 30 min. The resulting wear scars depth was measured using an Olympus laser confocal scanning microscope, and the wear rate was calculated. The mounted samples were polished and analysed using an Oxford scanning electron microscope with energy dispersive spectroscopy (EDS) and a Zeiss Optical Microscope. The compound phases formed at the treated surface of the titanium alloy were determined using a Bruker X-ray diffractometer.

**Table 1:** Parameter setting for the TIG process used to melt the surface of the Titanium alloy.

TIG welding parameters				
Welding type	Current	Torch angle	Distance between platform-torch	Platform moving speed
TIG parameters	75 Amp	45 degree	2 mm	2 mm/s
**Coating parameters**				
Bath 1 (S1)	250 g/L NiSO_4_.6H_2_O, 45 g/LNiCl_2_.6H_2_O, 35 g/L H_3_BO_3_, 1 g/L Saccharin, 1L H_2_O		40 nm Al_2_O_3_ and 250 nm TiO_2_ particles	20 g/L–Al_2_O_3_
				20 g/L–TiO_2_
Bath 2 (S2)	250 g/L NiSO_4_.6H_2_O, 45 g/LNiCl_2_.6H_2_O, 35 g/L H_3_BO_3_, 1 g/L Saccharin, 1L H_2_O		40 nm Al_2_O_3_ and 30 nm TiO_2_ particles	20 g/L–Al_2_O_3_
				20 g/L–TiO_2_

### Shielding gas

Argon was used as the shielding gas to prevent oxidation of the weld pool and stabilise the electric arc. Additionally, the scientific literature shows that the type of shielding gas affects the weld properties, shape, size, fusion, and welding speed. In this experiment, PURESHIELD ARGON (ISO 14175–11-Ar) has been used as a shielding gas containing 99.9% of Ar.

## Results and discussion

### Microstructure of the treated surfaces

Figure 1B shows an example of the coating deposited onto the titanium substrate prior to the secondary treatment step. The coating thickness was controlled by setting the coating time constant and using the same coating parameters for all samples coated during step one. The grey particles observed in Fig. 1B were a mixture of Al_2_O_3_ and TiO_2_ distributed through the coating thickness confirmed by EDS analysis. A coating thickness of 100 µm was achieved on each sample. Evaluation of the interface between the substrate and the coating indicated the absence of porosity or cavity, suggesting good conformance of the coating to the substrate. However, the cross-section of the coating shows the agglomeration of the nanosized particles distributed through larger clusters in some areas (see Fig. 1C. The agglomeration of particles is believed to have occurred in the as-received powder or during the deposition process^19^. Particle clusters could negatively impact the coating properties and the nano-phase distribution during the treatment process's second stage. A particle cluster is presented in Fig. 1C and confirms that many nanoparticles agglomerated to form a 2.9 µm diameter cluster. Various particle sizes are visible in the SEM micrograph within the particle clusters. Previous studies on these materials suggested particle agglomeration occurred in the as-received powders^17,20^.

The second process involved melting and mixing, impregnating the surface with the coating material. Figure 1D shows the appearance of the surface after step 2. The light grey region is believed to be unmelted sections of the coating mixed into the surface of the substrate and, in some sections, immersed into the treated layer of the alloy. Figure 1E shows the variation in the surface texture of the sample, with the red-coloured regions representing high spots while blue regions representing lower depths. Analysis with the scanning confocal microscope recorded an average surface roughness (Ra) value of 3.7 µm.

**Figure 2:**
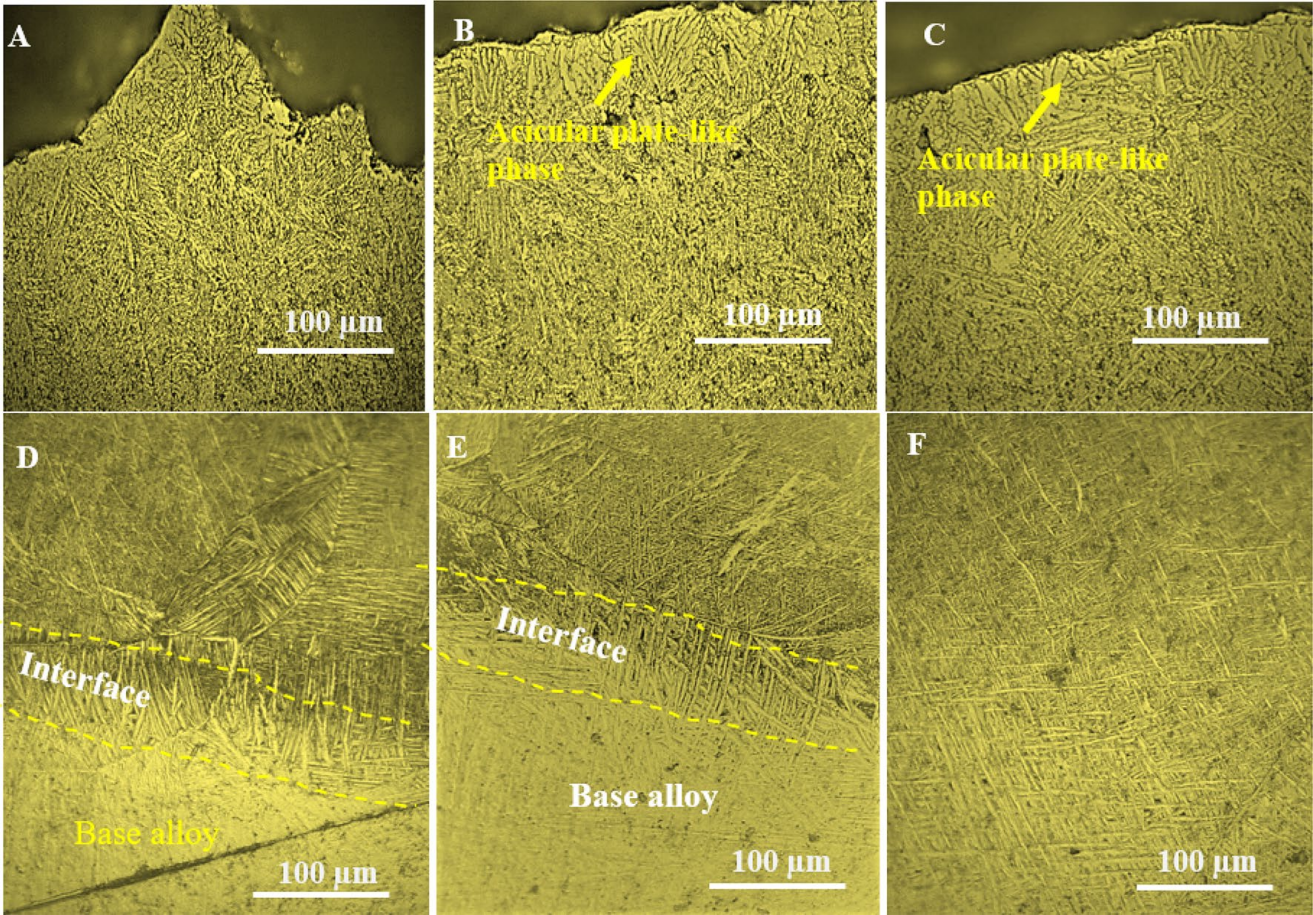
(**A**) Optical microscopic image of the microstructure formed (**A**–**C**) when 250 nm TiO_2_/40 nm Al_2_O_3_ and particles are deposited in the coating. (**D**–**F**) when 30 nm TiO_2_/40 nm Al_2_O_3_ particles are deposited in the coating.

The effect of TiO_2_ particle size on the properties of the treated layer was evaluated. The microstructure of the samples produced using a mixture of 30/250 nm TiO_2_ and 40 nm Al_2_O_3,_ co-deposited with Ni coating and surface melted using a TIG arc, is presented in Fig. 2. The sample coated with Ni containing 250 nm TiO_2_ and 40 nm Al_2_O_3_ developed a course acicular plate-like phase close to the sample's surface, with grain sizes increasing progressively into the sample towards the base metal (see Fig. 2A–C). The presence of a transition zone between the re-solidified layer and the bulk material shows the columnar grains' growth towards the melted layer. This confirms that the melted layer's solidification progressed inwards from the melted surface and outwards from the bulk material. Similarly, when the TiO_2_ particles sizes were reduced to 30 nm, the solidification of the treated layer progressed in the same way described above; however, the treated layers contained plate-like α crystals to acicular and Widmanstatten-like microstructure with very fine grains between the treated layer and the base alloy (see Fig. 2D–F).

**Figure 3:**
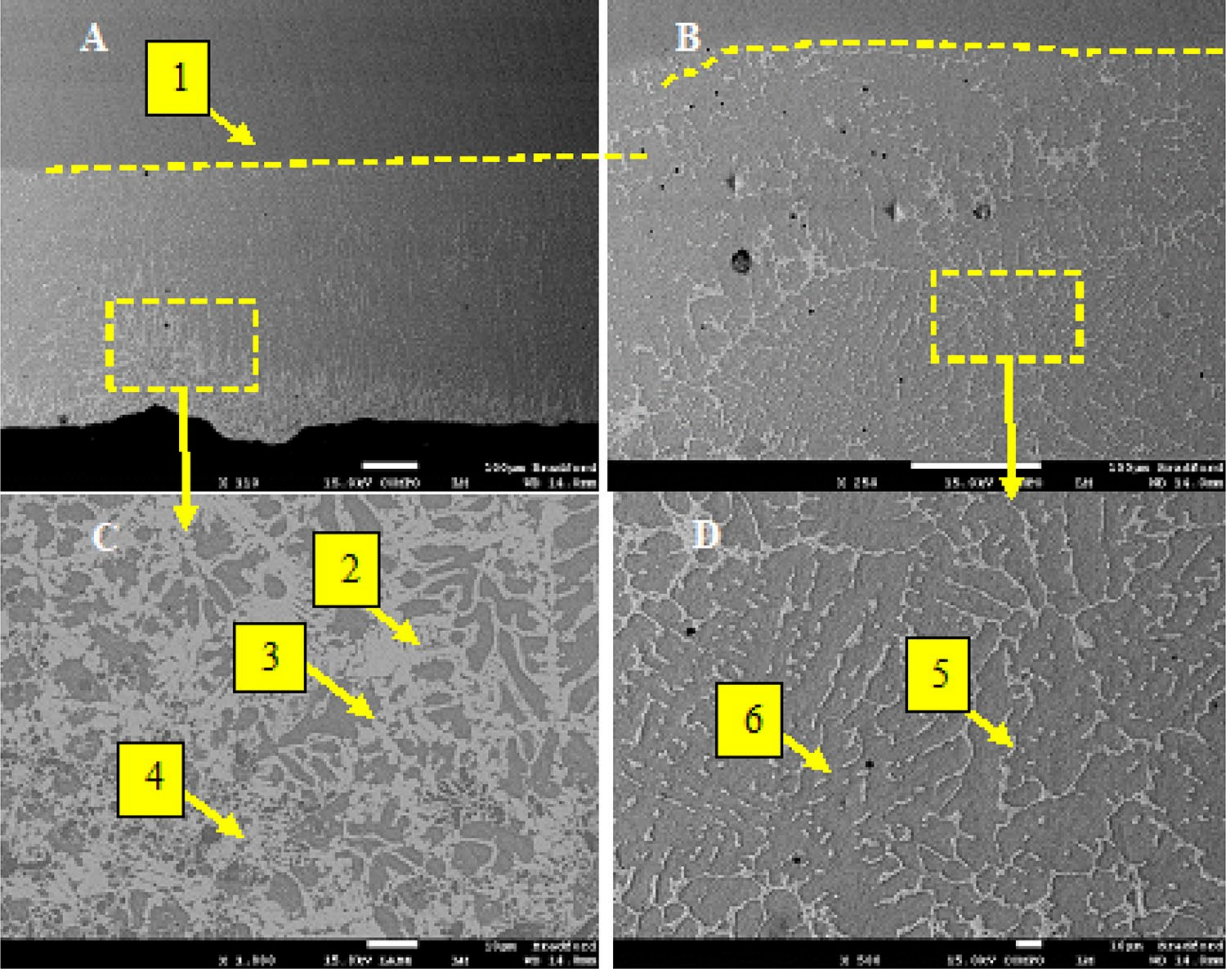
(**A**) The treated surface of the Ti-6Al-4V alloy 250 nm TiO_2_ and 40 nm Al_2_O_3_ (**B**) highlighted section of the treated layer (**C**) microstructure showing three distinct phases (**D**) Highlighted section of the treated zone showing the presence of large dark region labelled as 6 with the composition of τ_1_ = Al_13_Ni_2_Ti_5_ (Al_64_Ni_10_Ti_26_), and light colour region labelled as "5" which is likely of the composition τ2 =  = Al_2_NiTi (Al_55_Ni_23.5_Ti_21.4_).

**Figure 4:**
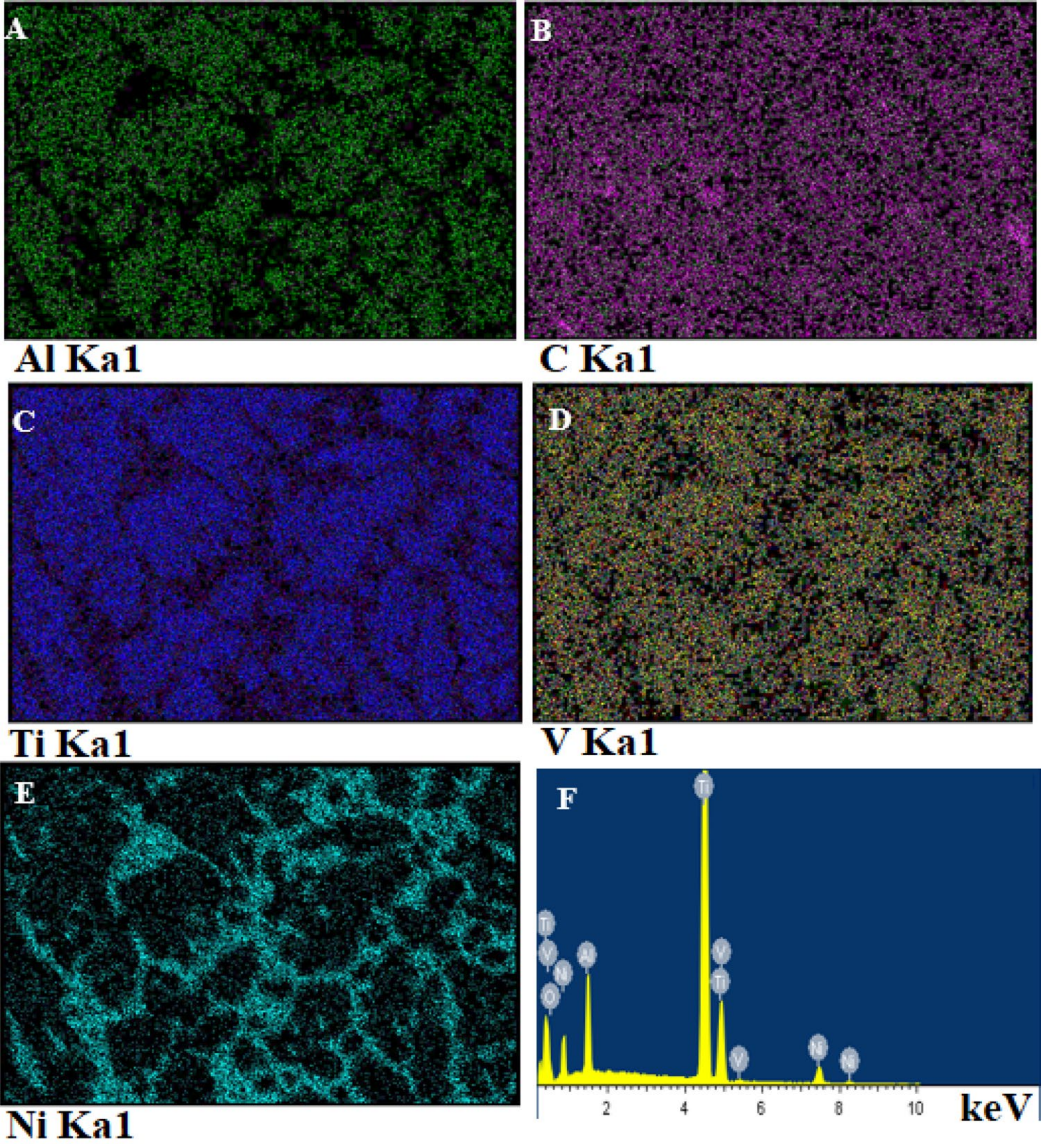
EDS map of treated Ti surface containing 250 nm TiO_2_ and 40 nm Al_2_O_3_ (**A**) Aluminum (**B**) Carbon (**C**) Titanium (**D**) Vanadium (**E**) Nickel (**F**) EDS spectrum of the treated zone.

The SEM micrograph of the Ti-6Al-4V surface impregnated with Ni containing 250 nm TiO_2_ and 40 nm Al_2_O_3_ is presented shown in Fig. 3. The treated layer of approximately 600 µm is visible in Fig. 3A and is clearly defined by a light grey shade containing several brighter regions. The region shown in Fig. 3B highlights the transition region between the treated layer and the titanium base metal. The light grey regions were found to have a high Ni concentration, as illustrated by the EDS map presented in Fig. 4E shows that Ni is the only element present in these areas, suggesting that the melted coating diffuses along the grain boundary. The nanoparticles appear to occupy a subsurface position in clusters pinpointed by label-4. Additionally, the EDS spot analysis presented in Table 2 indicates that the nickel content within the impregnated layer increase closer to the treated surface. The nickel appears to have diffused along grain boundary regions leading to the formation of a coarse microstructure.

**Table 2:** Chemical composition (wt%) of points 1–6.

Zone	Ni	Al	Ti	O	V	Potential major phases
1	3.55	5.33	87.43	–	3.63	τ_1_, τ_2_ , τ_3_ , τ_4_ or τ_5_
2	26.54	2.19	47.67	11.47	1.22	τ_1_, τ_2_ , τ_3_ , τ_4_ or τ_5_
3	6.63	3.41	59.02	18.52	2.66	τ_1_, τ_2_ , τ_3_ , τ_4_ or τ_5_
4	6.67	3.27	59.4	18.52	2.57	τ_1_, τ_2_ , τ_3_ , τ_4_ or τ_5_
5	26.54	2.19	47.67	11.47	1.22	τ_1_, τ_2_ , τ_3_ , τ_4_ or τ_5_
6	6.63	3.41	59.02	18.52	2.66	τ_1_, τ_2_ , τ_3_ , τ_4_ or τ_5_
7	–	5.05	74.05	1.93	2.97	TiAl
8	0	5.23	86.10	2.35	3.84	TiAl
9	9.85	4.03	82.29	–	3.83	τ_1_, τ_2_ , τ_3_ , τ_4_ or τ_5_
10	–	2.26	68.84	23.49	3.16	TiAl
11	–	5.23	85.10	2.35	3.84	TiAl
12	–	5.16	85.2	5.66	3.12	TiAl

During the surface melting process, the Ni/TiO_2_ /Al_2_O_3_ coating was melted and mixed into the surface of the titanium alloy. The constituents of the coatings appear to have been distributed through the treated layer within the grain boundary regions. The rapid solidification and subsequent quenching of the sample are believed to have resulted in a change in the grain structure leading to the formation of small grains within the treated layer. A high-magnification image of the surface is shown in Fig. 3C, confirming the presence of a network of small grains surrounded by nickel-rich layers in which the nano TiO_2_ and Al_2_O_3_ particles are distributed. The brownish/grey spots in the image are believed to be the nanoparticles expected to strengthen the material. Figure 3D shows the microstructure of the treated layer's centre, though this region is slightly different from the microstructure at the sample's surface as outlined in Fig. 3C; the melted nickel appeared to occupy the grain boundary regions still.

Figure 4 shows the EDS maps of the significant elements within the treated layer as point analysis. The distribution of Al, Ni and O confirm the presence of the essential elements deposited in the coating during step 1 and the mixture of Ni, Al and O in the melted zone. The elemental oxygen map shows the distribution of 250 nm TiO_2_ and 40 nm Al_2_O_3_ nanoparticles within the molten area. The grain structure within the treated layer changed to a much coarser microstructure with layer colonies of Ni-rich phases. The EDS maps presented in Fig. 4 illustrate Ni along the grain boundaries with nanoparticles distributed throughout the layer and confirm the size of the grains formed when larger particles are distributed in the coating.

**Figure 5:**
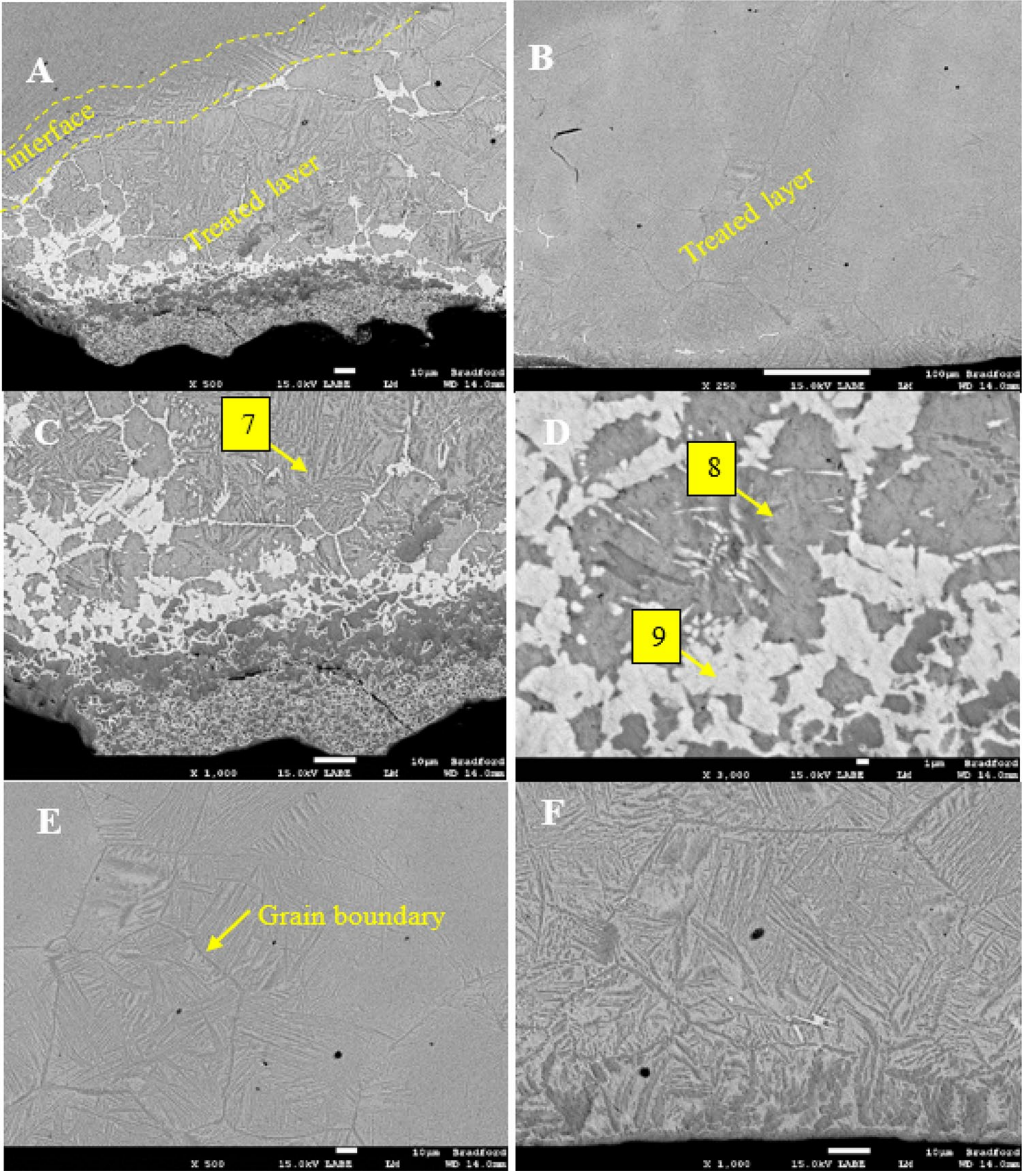
(**A**) The treated surface of the Ti-6Al-4 V alloy Ni coating containing 30 nm TiO_2_/40 nm Al_2_O_3_ (**B**) Highlighted.

Figure 5 shows the SEM micrographs of the Ti surface impregnated with Ni containing 30 nm TiO_2_ and 40 nm Al_2_O_3_ nanoparticles. The impregnated layer progressed to a 600 µm into the base metal (see Fig. 5A) and is made up of a transition zone/interface where the microstructure starts to change. The treated layer contains several regions, as denoted by the differences in shade. The light grey regions were identified by EDS analysis as nickel-rich zones predominantly in clusters approximately 40 µm below the surface Fig. 5D. Also, nickel appears to have diffused along the grain boundary, creating a microstructure distinct from the base metal. Figure 5C shows the sub-surface agglomeration of nanoparticles at approximately 10 µm below the free surface. Within the transition zone, fine needle-like grains are observed within more significant phase boundaries dominated by the diffusion of nickel. Three separate strengthening mechanisms are at play; solid solution strengthening due to the presence of Ni within the treated layer, dispersion strengthening due to the distribution of hard nanoparticles within the treated region and grain size reduction.

**Figure 6:**
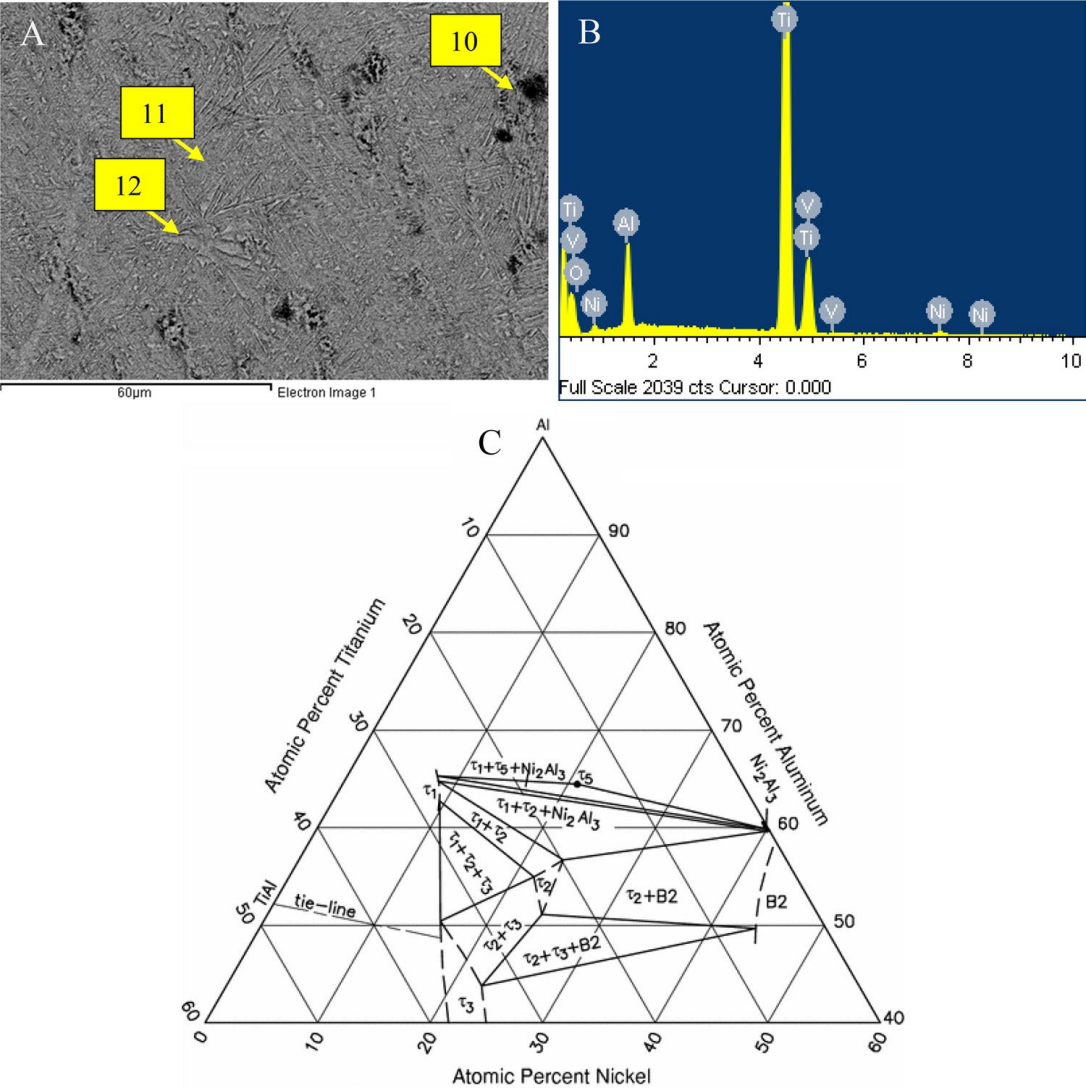
(**A**) SEM micrograph of the sample Ni coating containing 30 nm TiO_2_/40 nm Al_2_O_3_ (**B**) EDS spectrum of the treated layer showing the Ni content from the coating (**C**) Ti–Ni–Al phase diagram (V. Raghavan Al–Ni–Ti (Aluminum–Nickel–Titanium) *Journal of Phase Equilibria and Diffusion* **volume** **31**, pages 55–56 (2010)) .

EDS mapping of the transition region for samples 30 nm TiO_2_/40 nm Al_2_O_3_ shown in Fig. 6 confirms the distribution of Ni within the transition zone. The solution strengthening derived from the addition of Ni is expected to enhance the mechanical performance of the treated layer when compared to untreated Ti-6Al4V. Additionally, the dispersion strengthening of the nanoparticle will also enhance mechanical performance. While the nanoparticles were identified close to the free surface, they were not visible at depths greater than 10 µm, suggesting that the nanoparticles are most useful in reinforcing the immediate subsurface layer. Ten regions identified with yellow rectangles of the composition are presented in Table 2. Using the Ti–Ni–Al ternary phase diagram presented in Fig. 6, the formation of the following three likely phases TiAl, Ni_2_Al_3_, and NiAl. Additional ternary phases are also likely to form τ_1_ (Al_13_Ni_2_Ti_5_), τ_2_ (Al_2_NiTi), τ_3_ (AlNiTi), τ_4_ (AlNi_2_Ti), and τ_5_ (Al_65_Ni_20_Ti_15_).

**Figure 7:**
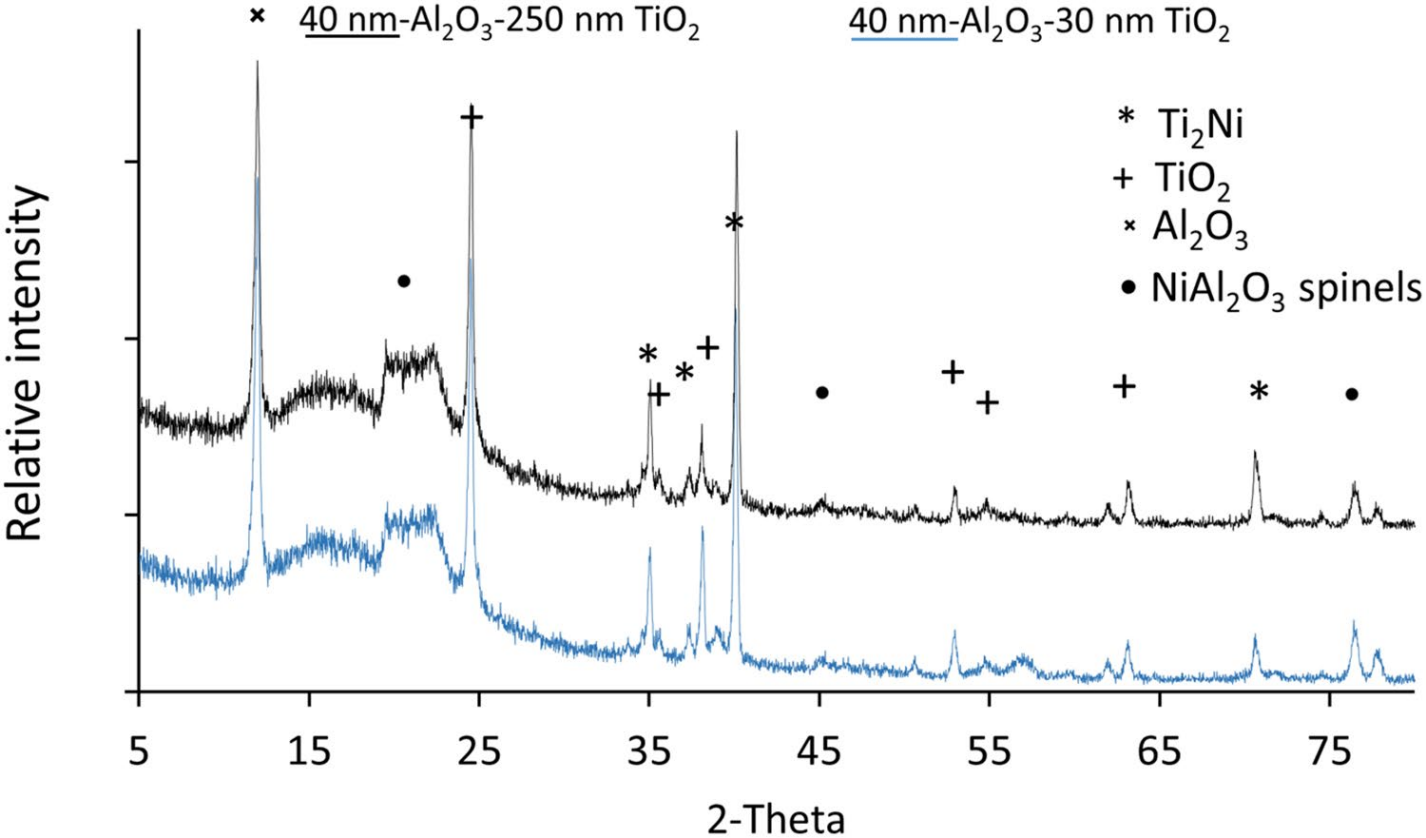
XRD analysis of the treated surfaces.

XRD analysis of the two treated surfaces to identify the main phases (see Fig. 7). The sharp peak indicates the presence of a crystalline structure which is also confirmed by the height of the peak present at 37° for samples coated with Ni containing 40 nm-Al_2_O_3_-30 nm TiO_2_. The hump present between 5 and 27° degrees suggests that the mixture consisted of both amorphous and crystalline phases. Alternatively, the broad peak may indicate smaller crystallite sizes in nanocrystalline materials, more stacking faults, micro-strain, and other defects in the crystal structure or an inhomogeneous composition in a solid solution. However, these peaks are not sufficiently broad to be conclusive of the role of the nano constituents within the impregnated layer.

### Hardness measurements

**Figure 8:**
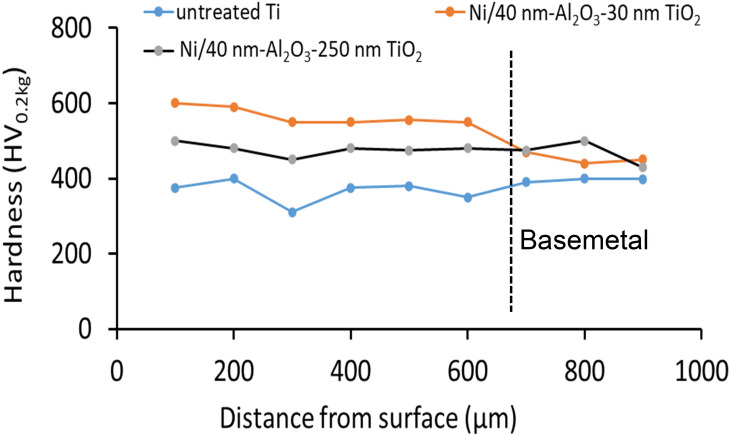
Hardness profile as function depth from the treated surface.

Mechanical characterisation of the impregnated layer provides the opportunity to isolate the impact of TiO_2_ particle size on the micro-hardness of the impregnated layer. The hardness profile presented in Fig. 8 compares the hardness profile of the two surfaces treated and the untreated titanium base metal as a control sample. The untreated basemetal appears to fluctuate at about 380 VHN, while higher hardness numbers are recorded for similar positions for the treated surfaces. The surface coated with Ni- containing the 40 nm-Al_2_O_3_-30 nm TiO_2_ mixture of nanoparticles produced surface hardness ranging from 600 HV_0.2 kg_ at the surface to 405 HV_0.2 kg_ at a depth of 900 µm. When compared to the surface coated with Ni- containing the 40 nm-Al_2_O_3_-250 nm TiO_2_ nanoparticles, the surface's hardness ranged from 500 HV0.2 kg to 398 HV_0.2 kg_ at a depth of 900 µm below the surface. Each indentation measured was performed at approximately 100 µm to prevent overlap of the stressed region surrounding the indentation.

**Figure 9:**
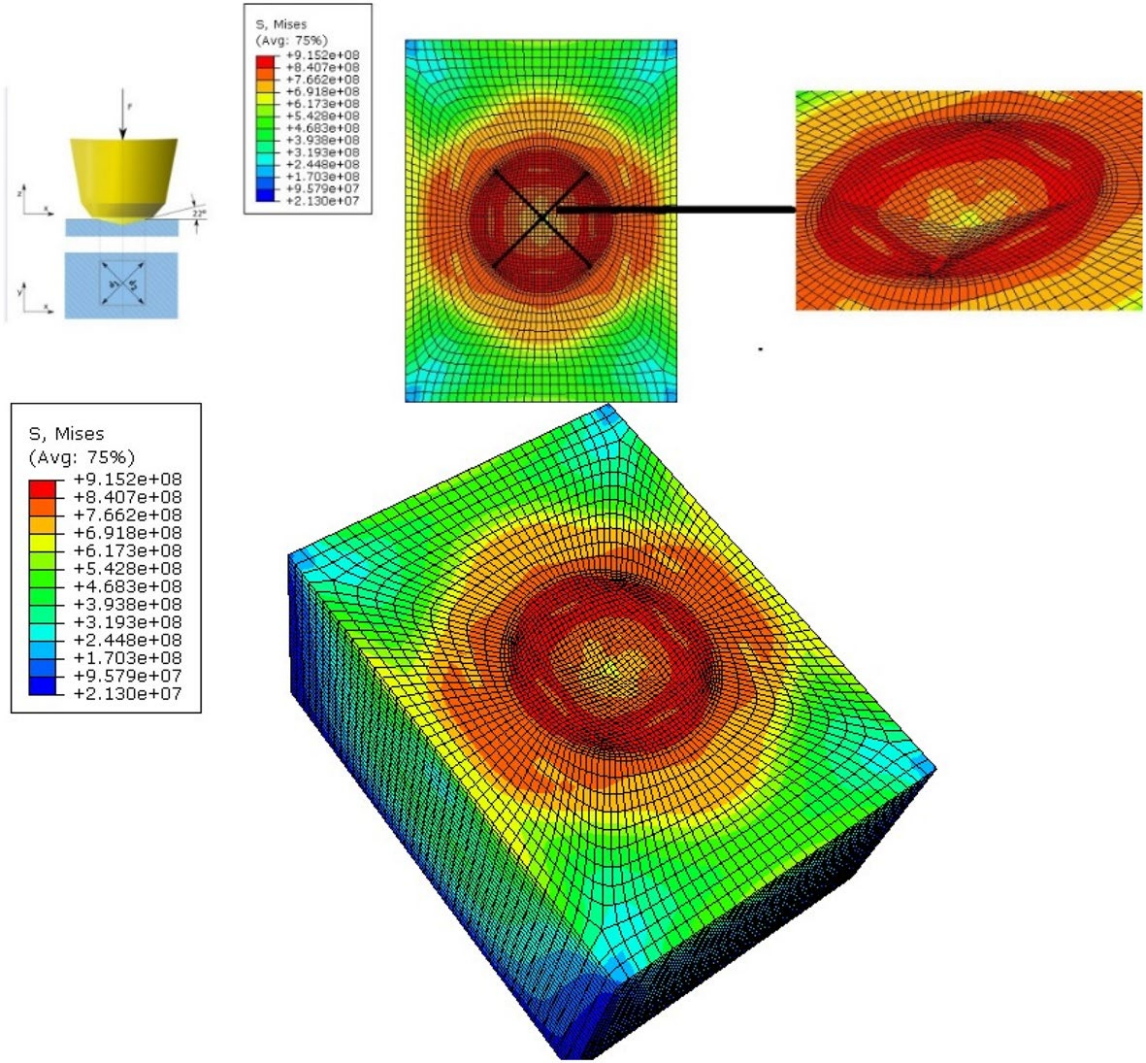
Modelling and simulation of the Vickers hardness test.

Figure 9 shows the stepwise simulation of the hardness testing and the size of the stress field generated during the hardness testing process. From the figure, it is clear that the stress start originating in the workpiece as soon as the tip of the tool touches the workpiece, and after the maximum impact, we can see clearly that the square helical-based impression is produced on the workpiece surface. Overlapping of the stress fields may result in work hardening of the region, causing a spike in the hardness number recorded.

The differences in the hardness of the treated and untreated surfaces were attributed to the impregnation of the surface with nanoparticles. When the nanoparticles' size was reduced, the hardness number increased, consistently. The scientific literature indicates that smaller dispersion particles are more effective at impeding dislocation motion, which is often reflected in an increase in the strength hardness of the material. Additionally, the reduction of the grain sizes due to modification of the microstructure within the treated layers positively impacts the hardness of the Ti-alloy. When the results generated are compared to other established processes, such as electroplating. This novel two-step process benefits become evident because electrodeposited coating tends to peel from the titanium surface^21^. The modified two step process produces a higher hardness than other coating techniques and is comparable to gas nitriding ^84^.

The principle of strengthening is quite complex and depends on the superposition of different strengthening mechanisms. The most common method is linear superposition, which can be simplified by isolating and adding the effect of each mechanism separately. Figure 9 shows the Von Mises stress field generated during the hardness testing of the samples, with the red-coloured regions denoting highly stressed areas. The impregnation of the titanium surface results in the activation of several strengthening mechanisms. Using Eq. (1) Hollomon's law^22^ shown below, the grain boundary strengthening effect can be evaluated:

1 $$\sigma_{1} = K\varepsilon^{n}$$

where K depends on a grain boundary diffusivity distribution function and n is the strain rate. The dissolution and diffusion of Ni into the treated surface cause Solid solution strengthening, as predicted by Eq. (2)^22^.

The hardness of the treated surface can be attributed to factors such as a reduction in the grain size within the treated region due to quenching of the treated sample and can be predicted from a modified Hall–Petch relationship, which takes into consideration two additional sources of strengthening solution strengthen and dispersion strengthening due to the addition of hard nanosize particles which are distributed throughout the treated region

2 $$\sigma_{2} = \sigma_{o} + \frac{{k_{y} }}{{d^{1/2} }}$$

where $\sigma_{2}$ is the flow stress in the untreated Ti6Al4V sample, $k_{y}$ is the material constant, *d* is the grain size within the treated layer and $\sigma_{o}$ is the tensile strengthen of the Ti6Al4V alloy. In this study a Hall–Petch constant of 0.75 MPa m^1/2^ was used for pure titanium^23^. Finally, assuming a uniform distribution of the hard nanosize particles and intermetallic compounds formed within the treated region, the impact of the individual strengthening mechanisms can be superimposed to have a profoundly positive influence on the mechanical properties of the surface. Assuming uniform distribution of the hard nanosize particles within the treated region. This portion of the sample can be treated as a composite to which the rule of mixtures equation can be applied.

3 $$\sigma_{3} = V_{m} \sigma_{2} + V_{p} \sigma_{p}$$

Taking all components into consideration

4 $$\sigma_{c} = \sigma_{1} + \sigma_{2} + \sigma_{3}$$

5 $$\sigma_{c} = K\varepsilon^{n} + V_{m} \left( {\sigma_{o} + \frac{{k_{y} }}{{d^{1/2} }}} \right) + V_{p} \sigma_{p}$$

wheret ${\sigma }_{c}$ represents that equivalent flow stress (${\sigma }_{c})$. In this study, the values were selected from the literature shows that Ti6Al4V has a tensile strength of ${\sigma }_{o}=$ 1014 MPa. Taking ${k}_{y}=0.75 MPa \sqrt{m}$ and d = 0.5 µm. Taking the volume fraction of particles in the solution as 18.4 wt%, while ${\sigma }_{p}=$ 300 MPa, K = 575 MPa, n = 0.4, and $\varepsilon =1.5$ are estimated for pure titanium [1, 19, 20]. Using the values listed above, the sum total of the strengthening effects can be calculated to be 1562.55 MPa, Which confirms that changes observed in the hardness results.

### Wear measurements

**Figure 10:**
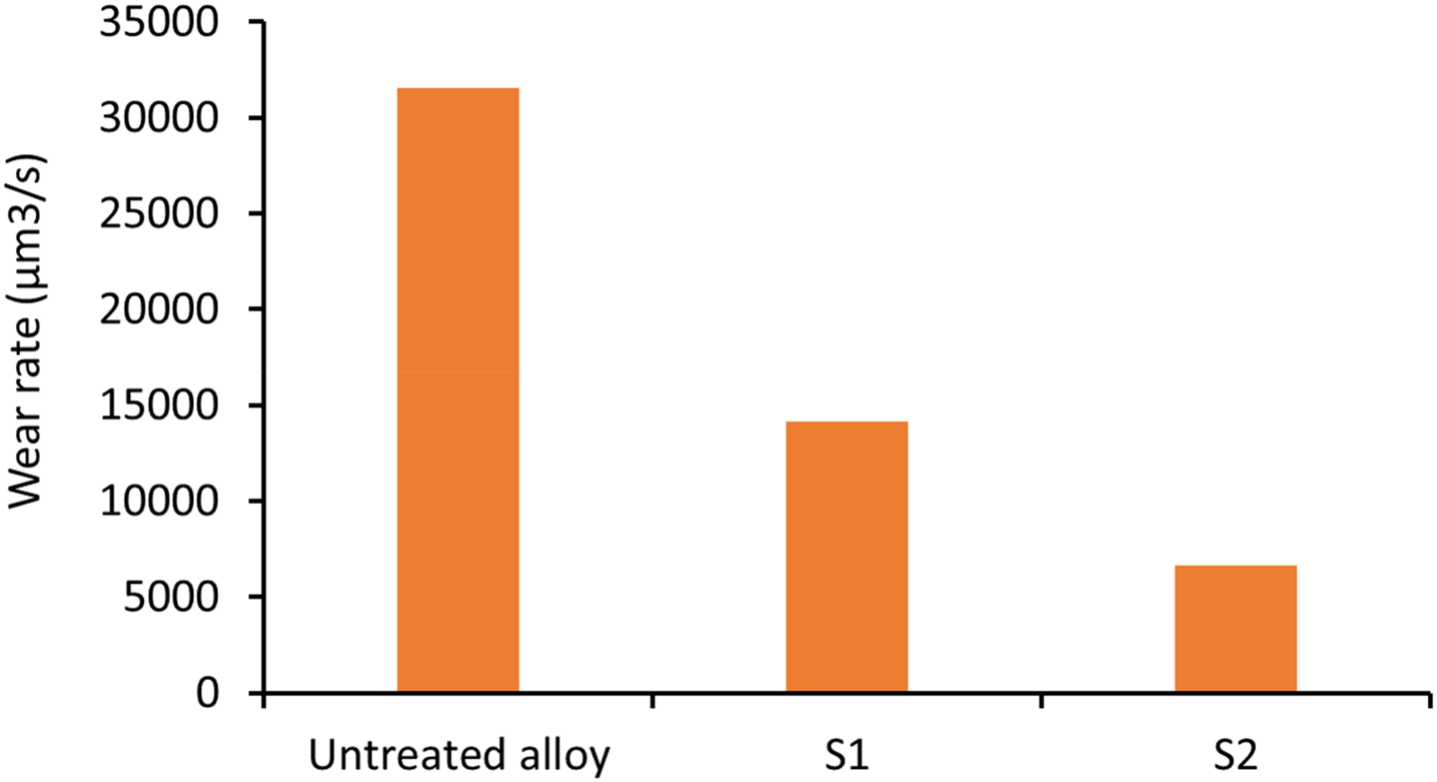
Wear rate of the samples tested. All samples were tested for thirty minutes S1-40 nm Al_2_O_3_-250 nm TiO_2_ and S2-40 nm-Al_2_O_3_-30 nm TiO_2_.

Figure 10 shows the results of the wear tests that were performed using a custom pin-on-plate wear test. The data shows that the wear rate of the samples was determined by measuring the volume of material removed from the wear scar using a scanning laser confocal microscope. Two groups of samples were prepared from the coating solutions. Samples prepared from bath-1 was labelled as S1 and samples prepared from bath-2 were labelled as S2. Sample S2 experienced the lowest scar depth and volume of removed materials. Both S1 and S2 provided better wear resistance than the untreated Ti-6Al-4V sample. The treated surfaces' mechanical performance can also be related to the distribution of nanoparticles deposited in the coating layer and their subsequent integration into the treated surface and the nanoparticles' size. This shows that smaller particles coherent with the lattice are more effective in strengthening the material. When the hardness of the treated layer is considered in the context of wear resistance, the literature shows that higher hardness of the protective layer is synonymous with higher wear resistance as predicted by Archard wear equation $\left( {Q = K\frac{WL}{H}} \right)$. The equation though simplistic in its approach, establishes a fundamental relationship between the volume of material (Q) removed during the wear process, the applied load (W), the Sliding distance (L) and the hardness of the material (H). The results confirm that if the sample's surface can be enhanced, then the wear resistance of the treated layer also increases.

**Figure 11:**
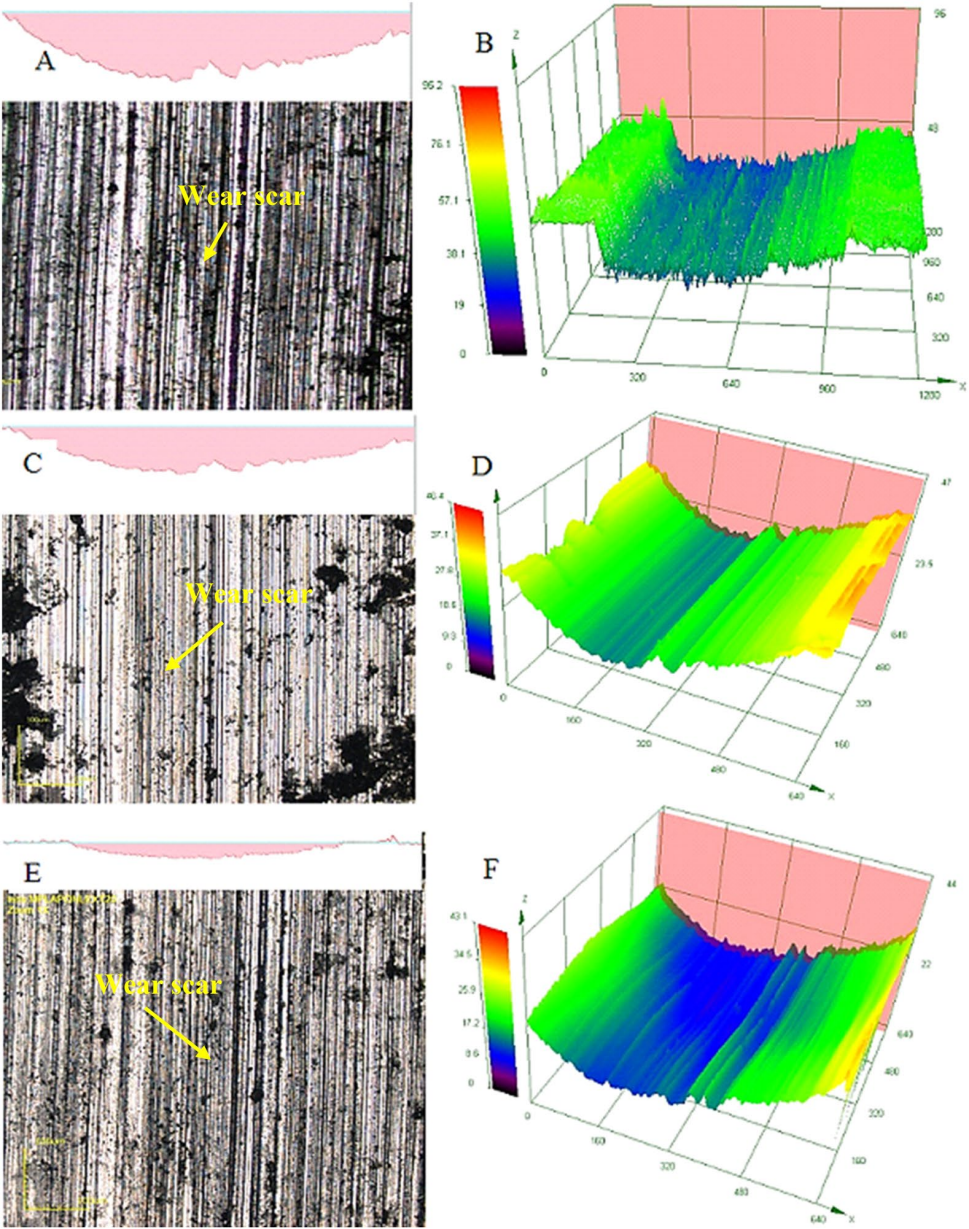
Wear track for untreated Ti-6Al-4 V sample (**A**) depth of the wear scar for untreated titanium alloy (**B**) Confocal topographical map of the wear scar for untreated titanium alloy (**C**) depth of the wear scar for the sample treated with S1-40 nm Al_2_O_3_-250 nm TiO_2_ (**D**) Confocal topographical map of the wear scar for the sample treated with S1-40 nm Al_2_O_3_-250 nm TiO_2_ (**E**) depth of the wear scar for the sample treated with an S2-40 nm-Al_2_O_3_-30 nm TiO_2_ (**F**) Confocal topographical map of the wear scar for treated titanium surface.

Several dark lines can be observed on the worn surface. The wear scars of the treated titanium surface and the untreated surfaces are shown in Fig. 11A. The results indicate a scar depth of 30 µm for the untreated titanium surface was produced over a 30-min testing time. The wear track of the untreated Ti-6Al-4V alloy is presented in Fig. 11, shows the presence of a series of parallel lines indicative of abrasive cutting and microploughing action of the diamond-tipped pin used in the wear testing process.

On the other hand, when the treated surface was tested, a wear scar of 8.7 µm was recorded at the wear scar centre, as shown in Fig. 10. The differences in the samples' depth were attributed to the increased hardness of the treated sample compared to the untreated surface. The embedding of nanoparticles into the titanium alloy's surface during the melting enhances the hardness and wear resistance of the treated layer by creating barriers to the fast dislocation movement. The modified microstructure of the treated layer is due to the non-equilibrium cooling of the samples. Figure 11C and D presents the treated samples' surfaces. Like the untreated alloy, parallel lines are visible along with cavities distributed throughout the sample. These cavities are believed to have been formed during the melting and mixing at the surface layer. When the wear testing was performed, the load applied during the process increased the cavities' size due to material removal. Larger holes are observed in the samples coated with the Ni containing 40 nm Al_2_O_3_-250 nm TiO_2_.

## Conclusions

In this work, we present a novel two-step process employed to harden the surface of Ti-6Al-4 V alloy. The sample preparation was achieved in two essential steps. The first step involved depositing a nanostructured Ni coating containing a mixture of Al_2_O_3_ and TiO_2_ nano-particles coating onto the surface of a Ti-6Al-4V alloy. The second step of the process involved using a TIG arc to melt the coated surface and a thin layer of the underlying basemetal. The results show that during the melting process, diffusion and mixing of the constituents into the treated layer led to the formation of various intermetallic compounds that contributed to an increase in the hardness of the treated surface compared to the untreated alloy. The hardness of the treated layer increased by more than 180% when 40 nm Al_2_O_3_ and 30 nm TiO_2_ particles were embedded into the surface. In both cases, the hardness of the treated layer was substantially higher than the hardness of the untreated Ti-6Al-4V alloy.

The results of the wear testing were consistent with the findings of the hardness test. Samples containing 30 nm TiO_2_ particles produced the most wear-resistant surfaces. Similarly, the wear resistance of the treated surface improved by 100%.

The microstructure analysis of the treated layers confirmed the microstructure variation from plate-like α crystals to acicular and Widmanstaten-like structures at the interface between the treated layer and the base alloy. The treated layer contained a higher Ni concentration close to the surface of the treated layer, with the nanoparticles distributed throughout. The presence of XRD peaks between 5° and 25° confirmed the presence of nanoparticles within the treated surface.

## Data Availability

The datasets used and/or analysed during the current study are available from the corresponding author on reasonable request.
